# HIV-1 Tat and Morphine Differentially Disrupt Pyramidal Cell Structure and Function and Spatial Learning in Hippocampal Area CA1: Continuous versus Interrupted Morphine Exposure

**DOI:** 10.1523/ENEURO.0547-20.2021

**Published:** 2021-05-19

**Authors:** William D. Marks, Jason J. Paris, Aaron J. Barbour, Jean Moon, Valerie J. Carpenter, Virginia D. McLane, Arianna R. S. Lark, Sara R. Nass, Jingli Zhang, Viktor Yarotskyy, A. Rory McQuiston, Pamela E. Knapp, Kurt F. Hauser

**Affiliations:** 1 Department of Pharmacology and Toxicology, Virginia Commonwealth University, School of Medicine, Richmond, VA 23298-0613; 2 Department of BioMolecular Sciences, University of Mississippi, School of Pharmacy, University, MS 38677-1848; 3 Department of Anatomy and Neurobiology, Virginia Commonwealth University, Richmond, VA 23298-0709; 4 Institute for Drug and Alcohol Studies, Virginia Commonwealth University, Richmond, VA 23298-0709

**Keywords:** drug abuse, hippocampal CA1 pyramidal neurons, learning and memory, neuro-acquired human immunodeficiency syndrome (neuroHIV), neuroadaptation to opioid exposure, synaptodendritic degeneration and dysfunction

## Abstract

About half the people infected with human immunodeficiency virus (HIV) have neurocognitive deficits that often include memory impairment and hippocampal deficits, which can be exacerbated by opioid abuse. To explore the effects of opioids and HIV on hippocampal CA1 pyramidal neuron structure and function, we induced HIV-1 transactivator of transcription (Tat) expression in transgenic mice for 14 d and co-administered time-release morphine or vehicle subcutaneous implants during the final 5 d (days 9–14) to establish steady-state morphine levels. Morphine was withheld from some *ex vivo* slices during recordings to begin to assess the initial pharmacokinetic consequences of opioid withdrawal. Tat expression reduced hippocampal CA1 pyramidal neuronal excitability at lower stimulating currents. Pyramidal cell firing rates were unaffected by continuous morphine exposure. Behaviorally, exposure to Tat or high dosages of morphine impaired spatial memory Exposure to Tat and steady-state levels of morphine appeared to have largely independent effects on pyramidal neuron structure and function, a response that is distinct from other vulnerable brain regions such as the striatum. By contrast, acutely withholding morphine (from morphine-tolerant *ex vivo* slices) revealed unique and selective neuroadaptive shifts in CA1 pyramidal neuronal excitability and dendritic plasticity, including some interactions with Tat. Collectively, the results show that opioid-HIV interactions in hippocampal area CA1 are more nuanced than previously assumed, and appear to vary depending on the outcome assessed and on the pharmacokinetics of morphine exposure.

## Significance Statement

Human immunodeficiency virus (HIV), transactivator of transcription (Tat), transgenic mice were co-exposed to morphine to explore opioid-HIV interactions in hippocampal area CA1. Spatial memory was impaired by both Tat and morphine. Tat expression reduced the firing rate of hippocampal CA1 pyramidal neurons at lower stimulating currents regardless of morphine exposure. Exposure to Tat and steady-state levels of morphine acted in a largely independent manner to alter pyramidal neuron structure, function, and associated behavior. This makes CA1 distinct from other regions such as the striatum. Alternatively, withholding morphine (from morphine-tolerant *ex vivo* slices) revealed unique, but subtle, neuroadaptive shifts in pyramidal neuronal excitability and dendritic plasticity, suggesting that opioid-HIV interactions in the hippocampus are markedly influenced by the pharmacokinetics of opioid exposure.

## Introduction

Human immunodeficiency virus-1 (HIV-1) infection results in neurocognitive impairments collectively termed HIV-associated neurocognitive disorders (HAND). Diminished spatial and verbal memory, frequently associated with hippocampal dysfunction, are hallmarks of HAND (Maki et al., 2009; Meyer et al., 2013; Keutmann et al., 2017). These and other deleterious HIV-related outcomes are aggravated by opiate abuse (Lucas et al., 2002; Denis et al., 2019). The opioid epidemic, which has partially driven the HIV pandemic (Nath et al., 2002; Campbell et al., 2017; Fitting et al., 2020), may impede current goals to eradicate HIV within 10 years (Fauci et al., 2019; Lerner and Fauci, 2019). Despite the prevalence of people infected with HIV (PWH) who are chronically exposed to opiates, the mechanisms underlying the neuropathological effects of HIV and morphine are incompletely understood.

The HIV-1 regulatory protein, transactivator of transcription (Tat), is intrinsically neurotoxic and has been shown to impair spatial memory in mice (Carey et al., 2012; Kesby et al., 2016; Marks et al., 2016). Morphine exerts similar antimnemonic effects (Zhu et al., 2011; Kitanaka et al., 2015). Previous work has identified HIV-1 Tat-induced reductions of long-term potentiation (LTP) in CA1 pyramidal neurons, which coincided with spatial memory deficits in Tat transgenic mice (Li et al., 2004; Fitting et al., 2013). This prior work showed only modest changes in synaptic spine density and no differences in excitatory synaptic protein content. However, Tat markedly reduced expression of synaptotagmin 2 (Syt2), while triggering presumably compensatory increases in gephyrin postsynaptically, suggesting losses in inhibitory GABAergic transmission within CA1 (Fitting et al., 2013). This selective vulnerability of interneurons within the CA1 may lower the threshold of excitation, leading to an increased likelihood of excitotoxicity. Dysregulation of excitatory/inhibitory tone may involve specific interneuronal subtypes and microcircuits (Fitting et al., 2013; Marks et al., 2016).

Opioid use disorder (OUD) can promote neurodegeneration within the hippocampus and other brain regions. Chronic abuse was found to accelerate Alzheimer’s disease-like pathology in multiple brain areas including the hippocampus of HIV-negative individuals in a cohort of preferential opiate misusers in Edinburgh, Scotland (Ramage et al., 2005; Anthony et al., 2010; Kovacs et al., 2015). This includes increases in hyperphosphorylated tau, GSK3β, and CDK5 levels, as well as microgliosis (Anthony et al., 2010). Within the hippocampus, opiate abuse alters CA1 ensemble activity (Liu et al., 2010) and impairs CA3-CA1 LTP (Borjkhani et al., 2018). Opiate abuse is linked to white matter damage (Li et al., 2016) and increased systemic inflammation (Piepenbrink et al., 2016). Even among PWH treated with combination antiretroviral therapy (cART), OUD (which often includes cocaine co-exposure) worsens HAND symptomatology, including deficits in verbal and working memory (Byrd et al., 2011, 2012; Meyer et al., 2013) and decision-making (Vassileva et al., 2013). Within the microcircuitry of hippocampal subregions, opiates and Tat may exert unique interactions. Morphine generally decreases interneuron excitability (Liao et al., 2005; McQuiston, 2008; Xu et al., 2013; Fitting et al., 2015); however, morphine’s capacity to inhibit GABAergic interneuronal inputs onto pyramidal cells may also exacerbate Tat-induced excitotoxicity. These outcomes are dependent on neuronal type and opioid receptor distribution (Drake and Milner, 2002; Liao et al., 2005; McQuiston, 2007, 2008; Xu et al., 2013).

OUD can exacerbate HIV-induced neuropathology across multiple brain regions (Fitting et al., 2010a, 2014, 2020; Hauser et al., 2012; Hahn et al., 2016), although because of the regional differences in the development of HIV-induced pathology and μ-opioid receptor (MOR) expression, each brain region is anticipated to display unique pathologic interactions and rates of decline (Fitting et al., 2010b). Given the Tat-mediated deficits previously observed in the GABAergic network of the mouse hippocampus, we hypothesized that the expression of Tat would disrupt the functional output of CA1, and that morphine would exacerbate these effects. We further expected that the reduction of Syt2 in the stratum radiatum (SR) layer of CA1 would result in a loss of synaptic contacts on pyramidal cells. To test these hypotheses, control and Tat transgenic mice were implanted with subcutaneous vehicle or morphine (25 mg) time-release implants. Mice were assessed for effects on spatial memory and motor behavior. Opioid injection drug use is accompanied by fluctuating drug levels (Kreek, 1987, 2001; Kreek et al., 2002) resulting in three to four bouts of “relative withdrawal” per day between injections (Reisine and Pasternak, 1996). To begin to assess the initial pharmacokinetic consequences of opioid withdrawal, morphine was withheld from some *ex vivo* slices during recordings. CA1 pyramidal cell function was studied via whole-cell patch-clamp electrophysiology in *ex vivo* slices in the presence or absence of morphine. Patched pyramidal cells were then visualized via biocytin backfill and 3D reconstruction, and morphology was assessed.

## Materials and Methods

The use of mice in these studies was preapproved by the Institutional Animal Care and Use Committee at Virginia Commonwealth University. Experiments were conducted in accordance with ethical guidelines defined by the National Institutes of Health (NIH Publication No. 85–23).

### Subjects and housing

This study used male mice between 8 and 12 weeks of age with or without the presence the HIV-1 *tat* transgene (HIV-1 Tat_1-86_) under the control of a doxycycline (Dox)-activated Tet-on expression system driven by a glial fibrillary acidic protein (GFAP) promoter (Bruce-Keller et al., 2008). Dox was administered in a specially formulated diet (Dox Diet #2018, 6 g/kg, Harlan Laboratories) to the mice for 14 d, after which tissues were harvested for biochemical or electrophysiological experiments. All mice were housed four to five per cage and maintained in a temperature-controlled and humidity-controlled room on a 12/12 h light/dark cycle (lights off at 6 P.M.) with food and water available *ad libitum*. On day 5 of Dox administration, vehicle or morphine pellets (25-mg morphine sulfate, NIDA Drug Supply System) were implanted subcutaneously.

### Surgical procedure

Subcutaneous vehicle-containing or morphine-containing pellets were implanted in Tat− (*n_vehicle_* = 17, *n_morphine_* = 15) and Tat+ (*n_vehicle_* = 18, *n_morphine_* = 15) mice under isoflurane anesthesia (2.5–4%) as described previously (Fitting et al., 2016). Following surgery, mice were monitored for 96 h to ensure weight gain, muscle tone, proper neurologic responses, and general health (Crawley and Paylor, 1997). All mice in the present study recovered.

### *Ex vivo* slice preparation for electrophysiological experiments

Following 14 d of Dox exposure, adult male mice were over-anesthetized with isoflurane anesthesia (5%), euthanized, and transcardially perfused with sucrose cutting media (3 mm KCl, 4.12 mm MgSO_4_, 1.2 mm NaH_2_PO_4_, 206 mm sucrose, 25 mm NaHCO_3_, and 25 mm glucose) chilled to 1–3°C and bubbled with a 5% CO_2_ balanced oxygen mix. Brains were dissected, bisected midsagittally, and 350-μm-thick, horizontal sections were cut from the ventral surface using a Leica VT1200 S vibratome (Leica Biosystems). Slices were cut in oxygenated sucrose cutting media held at 1–3°C by an external cooling apparatus (Huber), then transferred onto a nylon mesh submerged in oxygenated extracellular recording solution (3 mm KCl, 1.2 mm CaCl_2_, 1.2 mm MgSO_4_, 1.2 mm NaH_2_PO_4_, 125 mm NaCl, 25 mm NaHCO_3_, and 25 mm glucose) and maintained at 36.5°C for 30 min. In slices in which morphine is maintained (not withheld), the cutting solution and extracellular solution were supplemented with 500 nm morphine sulfate. The beaker was then returned to room temperature and the slices allowed to rest for 30 min before recording. In parallel experiments, morphine sulfate was withheld from the cutting and extracellular solutions to model acute (2–4 h) withdrawal in morphine-pelleted mice.

### Electrophysiological recording

Slices were continuously perfused with extracellular recording solution warmed to 30–34°C with an external heating apparatus (Warner Instruments, TC-344B). The CA1 subfield of the hippocampus was visualized using a 4× magnification objective on a Zeiss Axio Examiner A1 microscope (Zeiss). Magnification was switched to a 63× fluid-immersion objective to identify putative pyramidal neurons in the CA1 pyramidal layer. Pipettes for whole-cell patch-clamp physiology were pulled (Narishige PC-10 pipette puller; Narishige) from borosilicate glass pipettes (WPI #1B1505-4, World Precision Instruments) to a resistance of 2–6 MΩ. Pipettes were filled with an intracellular solution containing 135 mm KMeSO_4_, 10 mm HEPES, 2 mm MgATP, 0.1 mm NaGTP, 8 mm NaCl, 0.1 mm BAPTAK_4_, and 0.2% biocytin (pH 7.25). Membrane potentials were recorded using a MultiClamp 700B amplifier (Molecular Devices), processed using a Digidata 1550A digitizer, and analyzed using Clampex 10.4 software (Molecular Devices) on a Microsoft Windows-based computer. Membrane potentials were observed in response to stepwise, 25-pA current increases from −100 to 400 pA.

### Histologic processing of biocytin-filled pyramidal cells

After completion of electrophysiological recordings, slices containing biocytin-filled cells were moved into a 24-well plate and fixed with 4% paraformaldehyde in 1× PBS for 4–7 d at 4°C. Following fixation, slices were rinsed in PBS six times for 10 min each on a rocking platform at 4°C. Slices were permeabilized for 30 min in a solution of 50% ethanol in 1× PBS, containing 0.02% Triton X-100, then transferred into a similar solution containing 70% ethanol for 30 min, and returned to 50% ethanol for 30 min. Slices were rinsed as described earlier, and then blocked in PBS containing 2% normal chicken serum, 0.02% bovine serum albumin, and 0.02% Triton X-100 for 30 min. Primary antibodies against gephyrin (goat polyclonal, 1:1000, Sc-6411, Santa Cruz) were applied, and slices were incubated for 48 h on a rocking platform at 4°C. The slices were rinsed as before and incubated in secondary antibodies (donkey anti-goat IgG-Alexa Fluor 488 1:500, Invitrogen A1055; goat anti-rabbit IgG-Alexa Fluor 647 1:500, Invitrogen A-21244), as well as an Alexa Fluor 594-conjugated streptavidin probe to label biotin (1:100, Invitrogen, S-32356) for 48 h, and then rinsed in PBS. Slices were incubated in Hoechst 33342 at room temperature (0.5 μg/ml in PBS; Invitrogen, H3570) for 10 min, rinsed in PBS, and mounted on slides using ProLong Gold Antifade reagent (Invitrogen, P36930).

### Imaging, 3D reconstruction, and analysis of biocytin-filled pyramidal cell dendrites

Z-stack 3D imaging of neurons was performed using a Zeiss LSM 700 at 20× (plan-apochromat 0.8 NA, M27) and 63× (plan-apochromat 1.40 NA oil immersion DIC, M27) magnification (Zeiss). Hoechst 33342 was visualized using a 405 nm laser with a SP 490-nm filter, Alexa Fluor 488 using a 488-nm laser and a BP 490- to 555-nm filter, and Alexa Fluor 594 using a 555-nm laser with the variable secondary dichroic (VSD) beamsplitter set at 585 nm. Z-stack data were reconstructed into a 3D image using Imaris Bitplane 7.6.4. Primary dendrites were defined as dendrites that protrude from the cell body, with dendritic order increased by every branching point. Dendritic spine analysis was performed by a blinded observer using Imaris Bitplane 7.6.4 and regions selected for analysis were 20–30 μm in length and were selected at a 5- to 10-μm distance from the previous branch point and at least 5 μm from the next branch point. Dendritic spine densities are reported as the average number of spines per 10-μm length of dendrite/cell. Spine morphology was analyzed using uncompressed Z-stacks. Spines were counted along 20- to 30-μm-long dendrite segments parallel to the plane of view. The dendritic spine density determinations of the vehicle versus the morphine-replete groups, and the vehicle versus the morphine-withheld groups, were performed separately by separate individuals. Accordingly, the spine densities in the vehicle (control) groups in the morphine-replete and morphine-withheld groups differ from one another.

### Quantification of spine subtypes using ImageJ

In brief, mushroom, stubby, and thin/filopodial subtypes were identified in fluorescent images by a blinded observer, as described previously (Schier et al., 2017). Dendritic spines along a ∼30 μm segment of dendrite parallel to the plane of the z-slice were counted. Dendritic segments were analyzed within the stratum oriens (SO), SR, and stratum lacunosum moleculare (SL-M) anatomic strata of backfilled CA1 pyramidal neurons. Data are expressed as number of spines/10-μm length of dendrite from individual cells.

### Quantification of inhibitory puncta

Analysis of inhibitory puncta associated with specific neurite segments was performed using the co-localization module of Imaris Bitplane (version 9.0). Briefly, background signal was filtered out of the 488-nm channel (gephyrin), and the signal from the 594-nm channel (streptavidin-tagged pyramidal cells) was used as a mask to create a co-localization channel for gephyrin positive puncta occurring within spaces occupied by filled pyramidal cells. The number of puncta was quantified along ∼10 μm of the identified neurite segment. Data are expressed as the number of gephyrin-labeled puncta per micrometer. Analysis of inhibitory contacts took place on the aspinous portions of the apical and basilar dendrites immediately adjacent to the cell body, as well as the spiny portions at the distal ends of dendrites terminating in SL-M, as these dendritic compartments have the greatest percentage of extant inhibitory contacts on a “representative” CA1 pyramidal cell (Megías et al., 2001).

### Stereology

The volume fraction (V_V_) of hippocampal areas CA1, CA2, CA3, and the dentate gyrus were assessed stereologically. Tat− and Tat+ (*n *=* *5–6) mice were administered vehicle or 25-mg morphine (data not shown) time-release pelleted implants, perfused with 4% PFA and frozen at −80°C. Frozen hippocampal sections were sectioned 40 μm thick in the coronal plane and stained with Hoechst 33 342 (1:20,000 in PBS, 8 min at room temperature). Left hemispheres were imaged at 10× magnification and montaged using a Zeiss Axio-Observer Z1 microscope with a motorized stage encoder and computerized tile reconstruction (Zeiss, Zen Black 2.3). V_V_ was estimated using point count analysis and a standardized grid overlay (per Cavalieri’s principle; West et al., 1991; Calhoun et al., 1998; Mouton, 2002, 2014).

### Barnes maze task

All behavioral testing was conducted in the presence of 70-dB white noise with mice habituated to the testing room for 1 h before assessments. Behavioral data were recorded and digitally encoded using an ANY-maze animal tracking system (Stoelting Co).

Mice were assessed for spatial learning and motor function via the Barnes maze (Barnes, 1979) as modified from previously reported methods (Marks et al., 2016). After a day of surgical recovery, mice were prehabituated to the maze (day 1) followed by 4 d of testing (two trials per day over days 2–5), and a 1-d reversal probe trial (two trials on day 6). Briefly, on day 1, mice were prehabituated to a random escape hole for 2 min, then were placed in the brightly lit center of the Barnes maze (91-cm diameter, 90-cm height, with 20 holes, each 5-cm diameter; Stoelting Co) and guided to the escape hole where they remained for 2 min. Lastly, mice were placed under a glass cylinder next to the escape hole and allowed to volitionally enter (3 min max. latency), or were guided in, and remained for 2 min. On testing days, mice were placed in the brightly lit center of the Barnes maze and allowed up to 3 min to find an open escape hole (escape-hole quadrant counterbalanced across testing groups). Mice that did not enter the hole were gently guided to the hole and allowed to remain for 2 min. On the final day of testing, a reversal probe trial was conducted such that the correct goal box was rotated 180° from its original position. The mean response of both trials was analyzed on each day for all mice. Shorter latencies to find the escape hole, a greater proportion of time spent in the correct quadrant of the maze, and fewer errors were considered indices of greater learning (Camara et al., 2013). Distances and velocities traveled were used as motor indices (Marks et al., 2016).

### Vision testing

All mice were tested for visual function following conclusion of the Barnes maze test (Wersinger et al., 2002; Marks et al., 2016). Briefly, mice were suspended ∼30.5 cm above a vertical ring-stand and were lowered with the ring-stand ∼5 cm from the left or right visual field (close enough to allow visual, but not whisker, contact with the ring-stand). The left and right visual fields were assessed for each mouse, with the starting side counterbalanced across groups. Visual responding was considered positive when mice reached with the forepaws for the rod when presented to both the left and right side. A response to only one visual field is considered a negative response. One male animal (Tat+/morphine) failed vision testing and was excluded from analyses.

### Statistical analyses

Electrophysiological measures were assessed via repeated measures ANOVA (firing frequency) or two-way ANOVA (intrinsic membrane properties) with current step as the within-subjects factor (0–400 pA), and tissue genotype (Tat− or Tat+) and drug treatment combination (vehicle-exposed tissues, morphine-exposed tissue in morphine-free solution, or morphine-exposed tissue in morphine-replete solution) as the between-subjects factors. A priori planned comparisons were conducted on all electrophysiological data with morphine-exposed Tat− and Tat+ tissues (maintained in either morphine-replete or morphine-withheld solution), compared with their respective vehicle controls, with Bonferroni corrections for multiple comparisons applied. The density of dendritic spines was assessed separately by spine type via two-way ANOVA with tissue genotype (Tat− or Tat+) and drug treatment combination (vehicle-exposed tissues, morphine-exposed tissue in morphine-free solution, or morphine-exposed tissue in morphine-replete solution) as the between-subjects factors. Fisher’s protected least significant difference (PLSD) *post hoc* tests were used to assess group differences following main effects. Interactions were delineated via simple main effects and main effect contrasts that were α corrected for multiple comparisons. Behavioral data were assessed via repeated-measures ANOVA with Barnes maze testing trial (testing days 1–4 and reversal probe) as the within-subjects factor, and both mouse genotype (Tat− or Tat+) and drug treatment (morphine or vehicle) as the between-subjects factors. Fisher’s PLSD *post hoc* tests were used to assess group differences following main effects. Interactions were delineated via simple main effects and main effect contrasts that were α corrected for multiple comparisons. Effect size measures (η^2^, Cohen’s *d*) are presented following omnibus inferential statistics and main effect contrasts, respectively. All analyses were considered significant when *p *<* *0.05.

## Results

### Morphine and Tat influence the electrophysiological properties of CA1 pyramidal cells

We used whole-cell patch-clamp physiology to examine the firing frequency and other electrophysiological properties of CA1 pyramidal cells, as they represent the focal point of inhibitory and excitatory processing in the region before projecting out toward the entorhinal cortex. To assess the effects of Tat and morphine on physiological function of CA1 pyramidal cells as the functional output of CA1, a planned comparison testing approach was applied to assess the statistical differences between six sets of conditions: Tat− and Tat+ vehicle-treated mice (*n *=* *13 cells from three mice, and 11–12 cells from three mice, respectively), Tat− and Tat+ morphine-treated mice in which the *ex vivo* slices were continuously maintained in 500 nm morphine-containing physiological solutions during recordings and referred to as “morphine replete” (*n *=* *19 cells from 4 mice, and 13 cells from 4 mice, respectively), and Tat− and Tat+ morphine-treated mice in which morphine was withheld from the *ex vivo* slices that were maintained in physiological solutions lacking morphine during recordings and referred to as “morphine withheld” (*n *=* *29 cells from 8 mice, and 22 cells from 7 mice, respectively). Tat− and Tat+ vehicle-treated mice were independently compared with morphine-replete groups or to morphine-withheld groups, but morphine-replete and morphine-withheld groups were not directly compared with one another.

**Figure 1.**
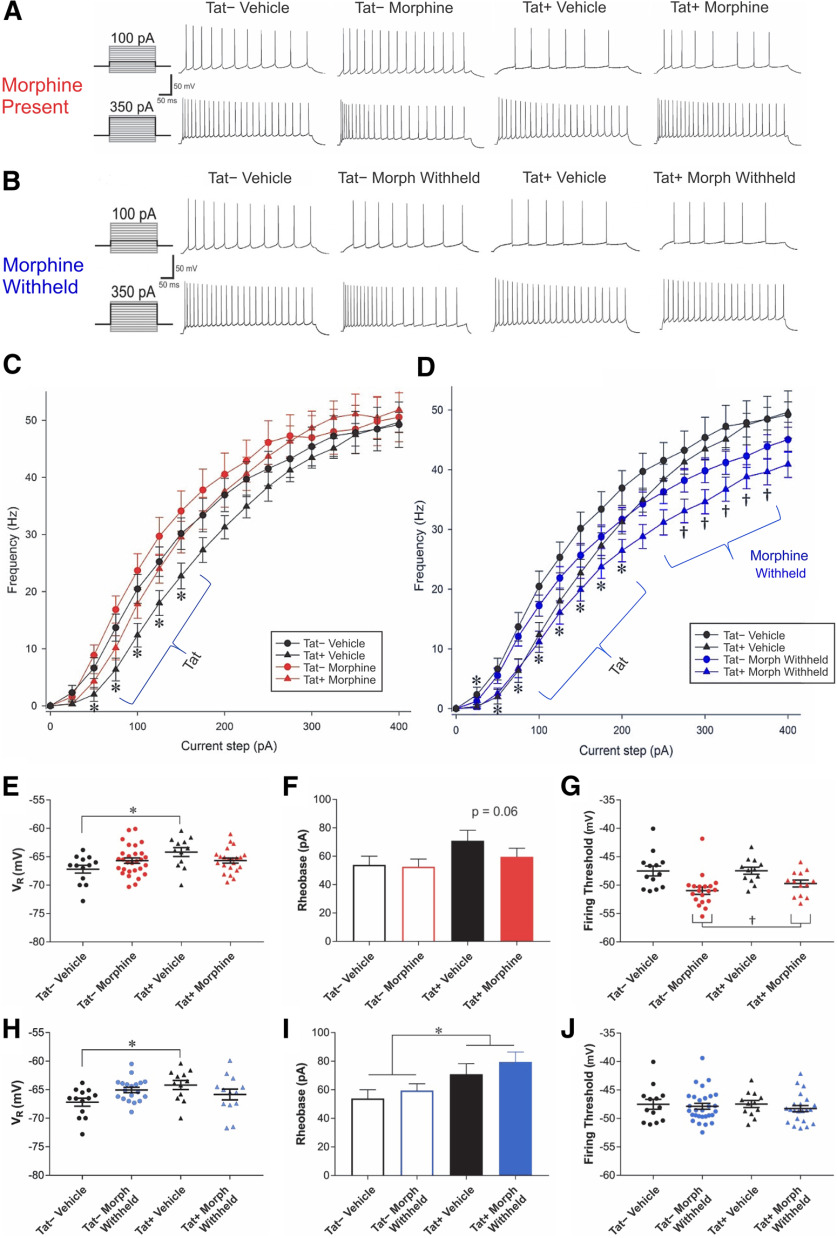
Analysis of the firing frequency of CA1 pyramidal cells from Tat– or Tat+ mice treated with vehicle or morphine time-release implants in which morphine is present (500 nm) or withheld (0 nm) during the recordings. ***A***, ***B***, Representative traces are depicted at the 100- and 350-pA current steps in which morphine (Morph) is present (500 nm; ***A***) or withheld (0 nm; ***B***) during the recordings. ***A***, ***C***, Pyramidal cell firing rates were unaffected by sustained morphine exposure. ***B***, ***D***, By contrast, when morphine was withheld during recordings from mice previously maintained on morphine, firing rates were significantly suppressed. ***C***, CA1 pyramidal cells from Tat+ mice fired at a lower frequency than those of Tat− mice between 50 and 150 pA, but were unaffected by exposure to morphine during recordings (***C***). ***C***, ***D***, Tat genotype interacted with the amount of current applied. ***D***, Withholding morphine from slices isolated from morphine-exposed mice resulted in significantly lower pyramidal cell firing rates at stimulating currents from 275 to 375 pA compared with morphine-naive cells (^†^*p *<* *0.05); nevertheless, Tat and morphine did not interact statistically perhaps because the effect of withholding morphine was not seen at other stimulating currents and at 25- to 200-pA current steps in pyramidal cells from Tat+ mice had lower firing rates than those from Tat− mice. ***E***, Resting membrane potential (V_R_) showed significant interactions when exposed to Tat or morphine. Tat+ neurons were significantly more depolarized at rest, while morphine treatment appeared to negate the effects of Tat. ***F***, While no significant effects were noted in rheobase, there was a trend (*p *=* *0.06) for pyramidal cells from Tat+ mice to require a greater amount of current to reach a threshold for firing compared with cells from Tat− mice. ***G***, The threshold for firing (mV) is significantly reduced in cells from morphine-treated mice. ***H***, There was a significant interaction between Tat and morphine on resting membrane potential (V_R_), which resulted from greater depolarized membrane potentials in vehicle-treated Tat+ mice. ***I***, Tat significantly increased rheobase, regardless of prior morphine treatment during the recording of morphine-withheld slices. Pyramidal cells from Tat+ mice required a greater amount of current (pA) to reach the threshold potential for firing than Tat− mice, although the firing threshold (mV) of CA1 pyramidal cells was unaffected by Tat or morphine exposure (***J***). Patched cells were stimulated with 500-ms current pulses starting at −100 pA and escalating to 400 pA in 25-pA steps; * indicates significant difference between Tat− and Tat+ tissues; ^†^ indicates a significant decline in firing rates in slices from Tat+, but not Tat−, mice previously exposed to morphine when morphine was withheld (*p *<* *0.05).

When comparing Tat− and Tat+ vehicle-treated mice to Tat− and Tat+ morphine-replete mice, several effects were observed (Fig. 1*A*,*C*,*E–G*). Repeated-measures assessment of firing frequency showed a significant interaction between current step and Tat genotype (*F*_(15,780)_ = 3.04, *p < *0.05; η^2^ = 0.007; Fig. 1*A*,*C*). *Post hoc* contrasts revealed that between the 50- and 150-pA current steps, pyramidal cells from Tat+ mice fired at a lower frequency than those from Tat− mice (*p *=* *0.002–0.046; Cohen’s *d *=* *0.5–0.8; Fig. 1*C*,*D*). Intrinsic membrane properties were assessed by two-way ANOVA. An interaction between Tat and morphine exposure was observed in the resting membrane potential of the CA1 pyramidal cells (*F*_(1,53)_ = 7.34, *p *<* *0.05; η^2^ = 0.12). Pairwise comparisons using the Bonferroni correction for multiple comparisons (significance threshold set at *p *<* *0.008) revealed that only vehicle-treated Tat− and Tat+ mice differed (Fig. 1*E*), with Tat+ vehicle-treated mice having significantly more depolarized resting membrane potentials (*p = *0.006; Cohen’s *d *=* *1.2). In addition, a main effect of morphine was observed on the firing threshold of CA1 pyramidal cells (*F*_(1,53)_ = 15.81, *p *<* *0.05; η^2^ = 0.22), with morphine-treated cells firing at a more hyperpolarized potential than vehicle-treated mice (*p *=* *0.001; Cohen’s *d *=* *0.5; Fig. 1*G*). No significant effects were noted for the smallest level of current required to elicit firing, or rheobase; however, Tat treatment tended to increase rheobase compared with Tat− controls (*F*_(1,53)_ = 3.37, *p *=* *0.062; η^2^ = 0.06; Fig. 1*F*). Notably, no significant differences in input resistance or capacitance were seen in pyramidal cells between any groups (Table 1).

**Table 1:** Membrane properties of CA1 pyramidal cells after exposure to Tat and morphine

*In vivo* treatment	Tat− Vehicle *n = *13	Tat+ Vehicle *n = *12	Tat− Morphine *n = *19	Tat+ Morphine *n = *13	Tat− Morphine *n = *29	Tat+ Morphine *n = *22
*Ex vivo* treatment	-	-	Morphine	Morphine	-	-
Capacitance (pF)	130.2 ± 12.4	175.4 ± 16.0	157.7 ± 9.5	138.4 ± 10.3	166.3 ± 8.8	184.7 ± 13.5
Resistance (MΩ)	182.1 ± 21.6	154.7 ± 16.9	146.4 ± 10.2	127.2 ± 8.9	179.0 ± 10.9	149.2 ± 16.2

Capacitance and resistance values from pyramidal cell recordings from Tat− and Tat+ mice maintained on vehicle (control)-containing or morphine (25 mg)-containing time-release implants in which morphine (500 nm) was present (“morphine”) or withheld (-) during recordings in hippocampal slices *ex vivo*. No significant changes were observed. All values are shown as the mean ± SEM.

**Figure 2.**
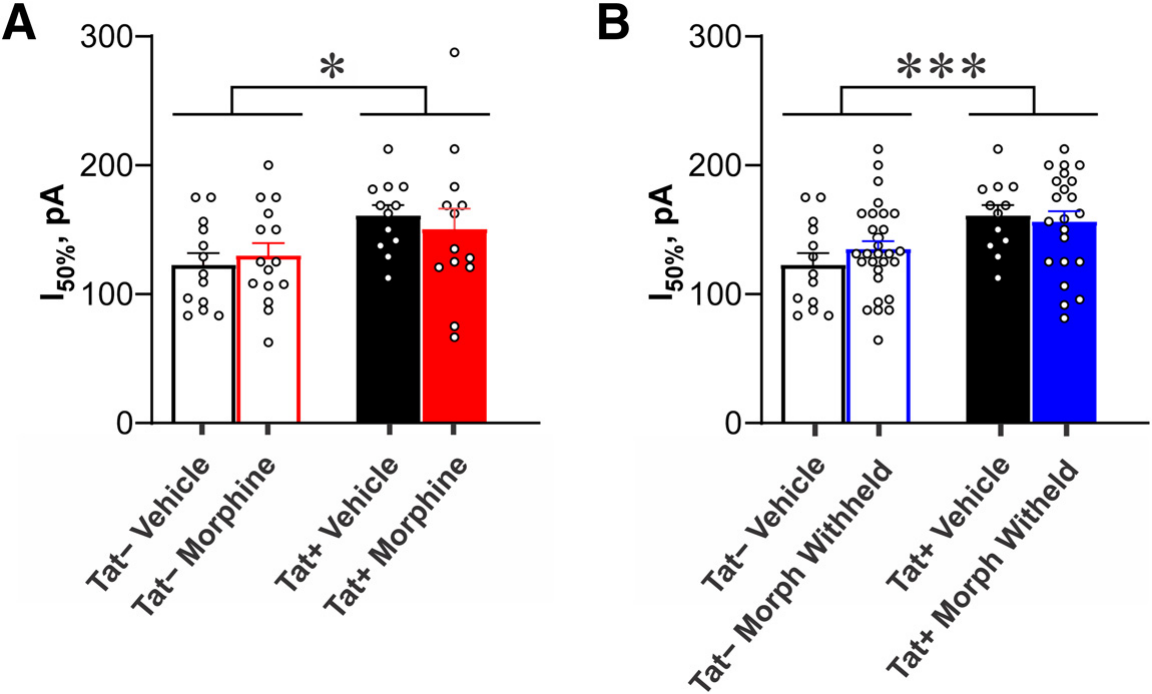
The amount of current injected to maintain I_50_%, or 50% of the maximal firing rate observed across all current steps (−100–400 pA in 25-pA steps) per cell (see Fig. 1*A–D*), is significantly increased in CA1 pyramidal cells of Tat+ compared with Tat− mice (main effect; *F*_(1,72)_ = 7.70, *p *<* *0.05), regardless of whether morphine is present (***A***) or withheld (***B***) during the recording. These data indicate that one week of Tat exposure is sufficient to fundamentally alter CA1 pyramidal cell excitability; * indicates a significant difference between pyramidal cells from Tat− and Tat+ mice (*p *<* *0.05); individual data points, representing individual cells, are shown by open circles.

To assess the effects of morphine withdrawal on the physiological response of CA1 pyramidal cells in mice previously exposed to morphine, vehicle-treated cells were compared separately to cells in which morphine (500 nm) was continuously present or absent (withheld) during the recordings (Fig. 1*B*,*D*,*H–J*). Analyses of the firing frequencies revealed two interactions, one between Tat and stimulus current amplitude (*F*_(15,1065)_ = 3.47, *p *<* *0.05; η^2^ = 0.004), and another between morphine treatment and stimulus current amplitude (*F*_(15,1065)_ = 6.43, *p *<* *0.05; η^2^ = 0.007; Fig. 1*B*,*D*). *Post hoc* contrasts revealed that Tat exposure altered the firing rate of pyramidal cells depending on the amount of the stimulating current. Tat+ pyramidal cells fired at a lower frequency than Tat− neurons (*p *=* *0.001–0.021; Cohen’s *d *=* *0.5–0.8; Fig. 1*D*) at 25–200 pA, although the firing rates did not differ at other current levels. When morphine was withheld from slices from morphine-treated mice, pyramidal cells fired at a significantly lower frequency than those in vehicle-treated mice when stimulated with 275–375 pA (*p *=* *0.007–0.02; Cohen’s *d *=* *0.6–0.7; Fig. 1*D*). Intrinsic membrane properties were compared using two-way ANOVA. An interaction between Tat and drug treatment was observed in the resting membrane potential (*F*_(1,72)_ = 37.99, *p *<* *0.05; η^2^ = 0.07). Pairwise comparisons using the Bonferroni correction for multiple comparisons (significance at *p *<* *0.008) revealed a significantly more depolarized resting membrane potential in pyramidal cells of Tat− versus Tat+ vehicle-treated mice (*p *=* *0.003; Cohen’s *d *=* *1.2; Fig. 1*H*). Additionally, Tat-exposed pyramidal cells required a greater amount of current (rheobase) to reach their firing threshold (*F*_(1,72)_ = 7.70, *p *<* *0.05; η^2^ = 0.10; Fig. 1*I*) and needed more current to attain their half-maximum firing frequency (I_50_%), regardless of whether morphine was present (*F*_(1,72)_ = 6.58, *p *<* *0.05; η^2^ = 0.73) or withheld (*F*_(1,72)_ = 4.95, *p *<* *0.05; η^2^ = 0.06; Fig. 2). No significant effects were observed on the firing threshold of CA1 pyramidal cells (Fig. 1*J*). As in the previous comparison, neither the resistance nor capacitance were significantly affected by Tat and/or withholding morphine during the recordings (Table 1).

### Morphine and Tat exposure alter the density and morphology of pyramidal cell dendritic spines within specific hippocampal layers in CA1

**Figure 3.**
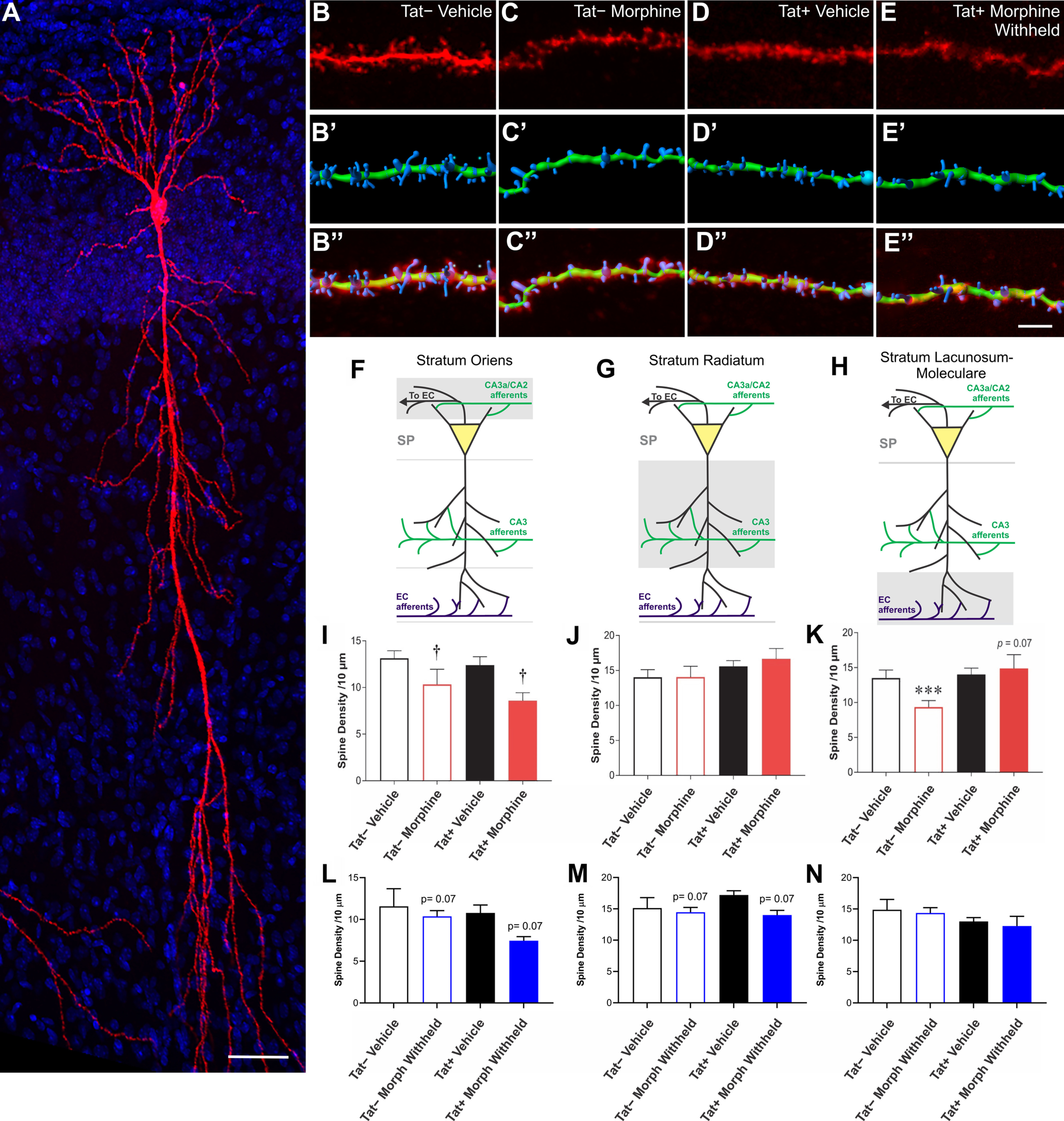
Effects of Tat and morphine on spine density along specific pyramidal cell dendritic segments within the SO, SR, and SL-M of hippocampal area CA1. ***A***, Reconstructed Z-stack image of a representative biocytin-filled pyramidal cell within hippocampal area CA1 from a vehicle-treated Tat− mouse. Scale bar: 50 μm. ***B–E***, Sample pyramidal cell dendritic segments and 3D reconstructions from the SO of control, morphine, Tat, and morphine plus Tat-exposed mice. The top image is the raw image file (***B–E***). The image below shows a representative 3D-reconstructed dendritic segment (green) and its associated spines (blue; ***B’–E’***). The final image superimposes the raw image of the dendrite and its spines with the 3D reconstruction of the same dendritic segment and spines (***B’’–E’’***). Scale bar: 10 μm. ***F***, ***I***, ***L***, Illustration of the location of pyramidal cell dendritic portion sampled is shaded in gray (***F***) and mean spine densities ± the SEM within the SO (***I***, ***L***). ***I***, There was a main effect of sustained morphine exposure *in vivo* and during recordings to reduce overall SO spine density (^†^*p = *0.007). ***L***, Spine losses were no longer significant after morphine was withheld, suggesting morphine-dependent spine losses are plastic and reversible. ***G***, ***J***, ***M***, Illustration of the location of pyramidal cell dendritic portion sampled is shaded in gray (***G***) and mean spine densities ± the SEM within the SR (***J***, ***M***). ***J***, No differences in spine density in SR dendrites were observed in Tat− or Tat+ in the absence or presence of sustained exposure to morphine *in vivo* and during recordings *ex vivo*. ***M***, By contrast, withholding morphine tended to induce spine losses. ***H***, ***K***, ***N***, Illustration of the location of pyramidal cell dendritic portion sampled is shaded in gray (***H***) and mean spine densities ± the SEM within the SL-M (***K***, ***N***). ***K***, Morphine caused a reduction in SL-M dendritic spine density in pyramidal cells from Tat−, but not Tat+, mice (***p <* *0.05). In fact, there was a trend for Tat to reverse morphine-dependent reductions in dendritic spine density with sustained morphine exposure *ex vivo*, albeit not significantly (*p *=* *0.07), suggesting that Tat and morphine uniquely interact to increase spine numbers on the SL-M (***G***). ***N***, By contrast, withholding morphine from *ex vivo* slices during recordings negated any changes in spine density seen when morphine is present (***N*** vs ***K***), suggesting the changes in spine density caused by morphine are highly plastic and modifiable.

Distinct subsets of interneurons that project to specific pyramidal cell dendritic subdomains in discrete hippocampal layers (Pelkey et al., 2017) are a key determinant in the inhibitory-excitatory balance in CA1 (Bourne and Harris, 2011; Hiratani and Fukai, 2017). Tat perturbs distinct subsets of CA1 interneurons (Marks et al., 2016), selectively depletes Syt2-immunoreactive fibers in the SR, and suppresses LTP (Fitting et al., 2013), which were predicted to alter spine density along specific dendritic subdomains (Fig. 3). The morphologic effects of Tat on CA1 pyramidal cells were previously analyzed as whole cells or larger segments without consideration to these laminar divisions (Fitting et al., 2013). To better assess the regional variations within CA1 dendritic structure, the neurons used in the electrophysiological analyses were filled with biocytin (Fig. 3*A*) and analyzed morphologically with respect to CA1 laminae (*n *=* *10–12; Fig. 3*B–J*).

The effects of Tat and morphine on the density of dendritic spines differed in each CA1 layer. In the continued presence of morphine, there was a main effect of morphine on spine density in the SO (*F*_(1,41)_ = 8.402, *p *<* *0.05; η^2^ = 0.17), where cells from morphine-treated mice had fewer spines per 10 μm segment (*p *=* *0.007; Fig. 3*I*). Spine density in the SR was not affected by either morphine or Tat (Fig. 3*I*). Spine density in the SL-M was unaffected by Tat; however, in morphine-replete pyramidal cells, there was a trend for Tat to interact with morphine to increase SL-M spine density (*F*_(1,38)_ = 3.47, *p *=* *0.07; η^2^ = 0.07). Consequently, the effects of morphine on SL-M spine density were examined further in pyramidal cells in Tat− and Tat+ mice separately. Significant effects were observed in Tat− cells (*t*_(19)_ = 2.823, *p *<* *0.05; η^2^ = 0.29), but not Tat+ cells. Morphine-treated pyramidal cells in Tat− mice had fewer SL-M spines than in vehicle-treated Tat− mice (*p *=* *0.015; Fig. 3*K*). Furthermore, there were differences in the density of dendritic spines depending on whether morphine was present or absent during the recordings. When morphine was withheld during the recording period, a trend toward reductions in spine density was evident in the SO (*F*_(1,38)_ = 3.64, *p = *0.064; η^2^ = 0.08; Fig. 3*L*) and SR (*F*_(1,38)_ = 3.55, *p = *0.067; η^2^ = 0.08; Fig. 3*M*), but not the SL-M, of slices from Tat+ mice that had previously been exposed to morphine.

**Figure 4.**
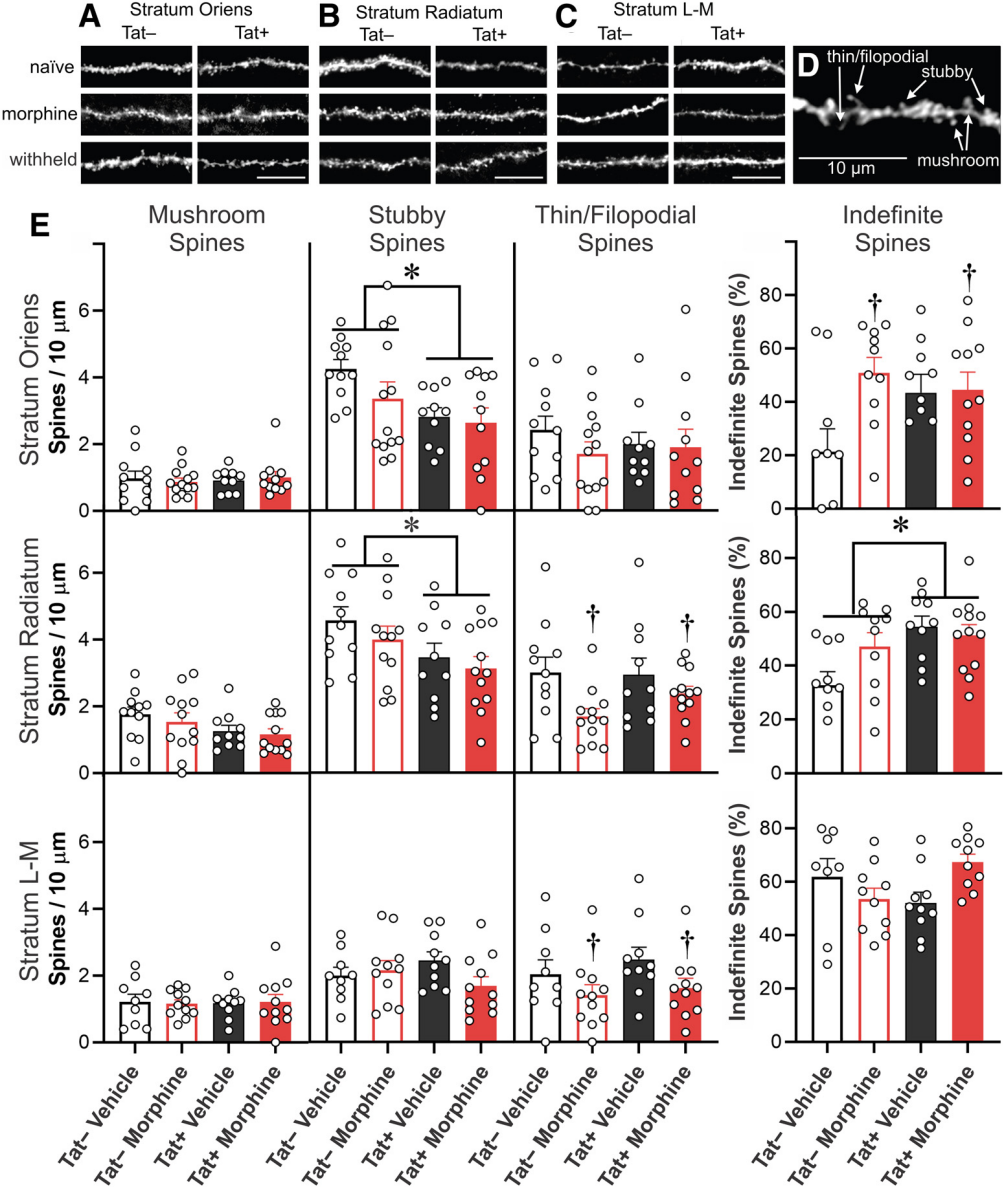
***A–E***, Spine subtype analyses of CA1 pyramidal neurons cells from Tat– or Tat+ mice. Mice were treated with vehicle control or morphine (25 mg) time-release implants; during electrophysiological recordings, morphine was present (500 nm, “morphine”). Spine subtypes were analyzed independently in SO (***A***), SR (***B***), and SL-M (***C***) according to their morphological classification as “mushroom,” “Stubby,” or “thin/filopodial” type spines (***D***). Dendritic spines of an indefinite or intermediate morphology were analyzed separately, thereby reducing the overall estimates of spine density compared with measurements in which all spines are counted (Fig. 3). Scale bars: 10 μm. (***E***) Tat exposure reduced intermediate, stubby dendritic spine subtypes in the SO and SR of morphine-replete pyramidal cells. There was a significant increase in the percentage of indefinite or intermediate spines in pyramidal cells in the SO with sustained morphine exposure. Within the SR, the proportion of indefinite or intermediate spines were increased in Tat-exposed pyramidal cells continuously exposed to morphine. Continuous morphine treatment decreased thin/filopodial spines in the SR and SL-M, while increasing SO indefinite spines in Tat exposed cells; * indicates a significant difference between Tat− and Tat+ tissues; ^†^ indicates a significant difference between morphine-replete and vehicle-treated tissues, *p *<* *0.05.

**Figure 5.**
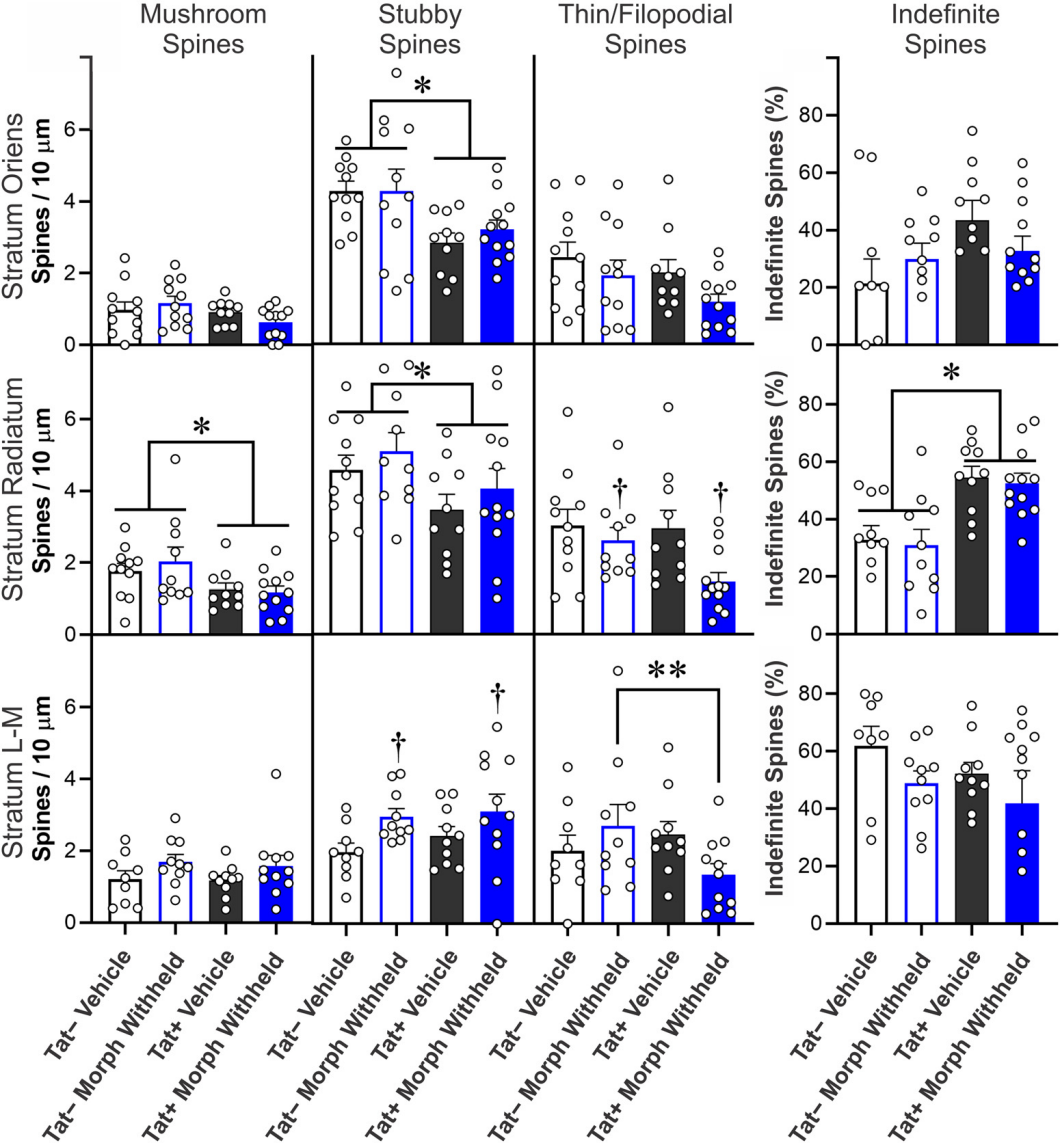
Spine subtype analyses of CA1 pyramidal neurons cells from Tat– or Tat+ mice. Mice previously treated with vehicle control or morphine (25 mg) time-release implants in which morphine was withheld (0 nm, “withheld”) during electrophysiological recordings. Spine subtypes (mushroom, stubby, or thin/filopodial) were analyzed individually. Dendritic spines of an indefinite or intermediate morphology were analyzed separately, thereby reducing the overall estimates of spine density compared with measurements in which all spines are counted (Fig. 3). Tat exposure reduced intermediate, stubby dendritic spine subtypes in the SO and SR of pyramidal cells in which morphine was withheld. Within the SR, withholding morphine increased the proportion of indefinite spines with concurrent Tat exposure. In the SL-M, morphine treatment increased the density of stubby spines when morphine was withheld, regardless of Tat exposure. Tat exposure reduced mushroom type spines in the SR regardless of treatment. Withholding morphine reduced SR thin/filopodial spine densities regardless of genotype. By contrast, withholding morphine interacted to significantly alter SL-M thin/filopodial spine density in Tat-exposed pyramidal cells, while withholding morphine specifically increased the proportion of thin/filopodial spines in Tat− mice; * indicates a significant difference between Tat− and Tat+ tissues; ^†^ indicates significant a difference between vehicle-treated and morphine-withheld tissues, *p *<* *0.05.

The morphology of dendritic spines transforms from more transient, immature thin/filopodial spines to mature mushroom spines as they become stable (Harris et al., 1992; Ochs et al., 2015; Schier et al., 2017). Representative images of dendritic spines within the major CA1 strata are shown in Figure 4*A–C*. To unambiguously categorize dendritic spines of a morphologic type, about one half of spines with indefinite or intermediate morphology were excluded; thereby reducing the overall estimates of spine density compared with measurements in which all spines are counted (Figs. 4, 5). Tat exposure reduced intermediate, stubby dendritic spine subtypes in the SO (*F*_(1,41)_ = 6.96, *p *=* *0.012; η^2^ = 0.14; Fig. 4*E*) and SR (*F*_(1,41)_ = 6.13; η^2^ = 0.06; *p *=* *0.018) of morphine-replete, as well as the SO (*F*_(1,40)_ = 10.77; η^2^ = 0.21; *p *=* *0.002; Fig. 5) and SR (*F*_(1,39)_ = 4.74; η^2^ = 0.11; *p *=* *0.036; Fig. 5) of pyramidal cells in which morphine was withheld (Fig. 5). There was a significant increase in the percentage of indefinite or intermediate spines in morphine-replete pyramidal cells in the SO (*F*_(1,37)_ = 4.72, *p *=* *0.0363; η^2^ = 0.10; Fig. 4*E*). Within the SR, the proportion of indefinite or intermediate spines were increased by Tat exposure in morphine-replete (*F*_(1,38)_ = 8.11, *p *=* *0.0071; η^2^ = 0.16; Fig. 4*E*) and morphine withheld (*F*_(1,38)_ = 22.85, *p *<* *0.0001; η^2^ = 0.38; Fig. 5) pyramidal cells. Within the SL-M, withholding morphine increased the density of stubby spines compared with vehicle, regardless of Tat exposure (*F*_(1,36)_ = 5.92; η^2^ = 0.02; *p *=* *0.020; Fig. 5). Tat-exposed pyramidal cells had reduced mushroom type spines in the SR, regardless of treatment in comparisons in which morphine was withheld (*F*_(1,39)_ = 7.37; η^2^ = 0.16; *p *=* *0.010; Fig. 5). Continuous morphine treatment decreased thin/filopodial spines in the SR (*F*_(1,41)_ = 6.96; η^2^ = 0.14; *p *=* *0.012; Fig. 4*E*) and SL-M (*F*_(1,37)_ = 4.50; η^2^ = 0.11; *p *=* *0.0406; Fig. 4*E*). Withholding morphine reduced SR thin/filopodial spine densities regardless of genotype (*F*_(1,39)_ = 5.78; η^2^ = 0.12; *p *=* *0.021; Fig. 5). By contrast, withholding morphine interacted to significantly alter SL-M thin/filopodial spine density only in Tat-exposed cells (*F*_(1,36)_ = 4.32; η^2^ = 0.10; *p *=* *0.045; Fig. 5), where withholding morphine in Tat− mice, but not Tat+ mice, significantly increased the proportion of thin/filopodial spines (*p *=* *0.029; Cohen’s *d *=* *0.9).

### Morphine and Tat exposure did not alter the density of inhibitory postsynaptic puncta

**Figure 6.**
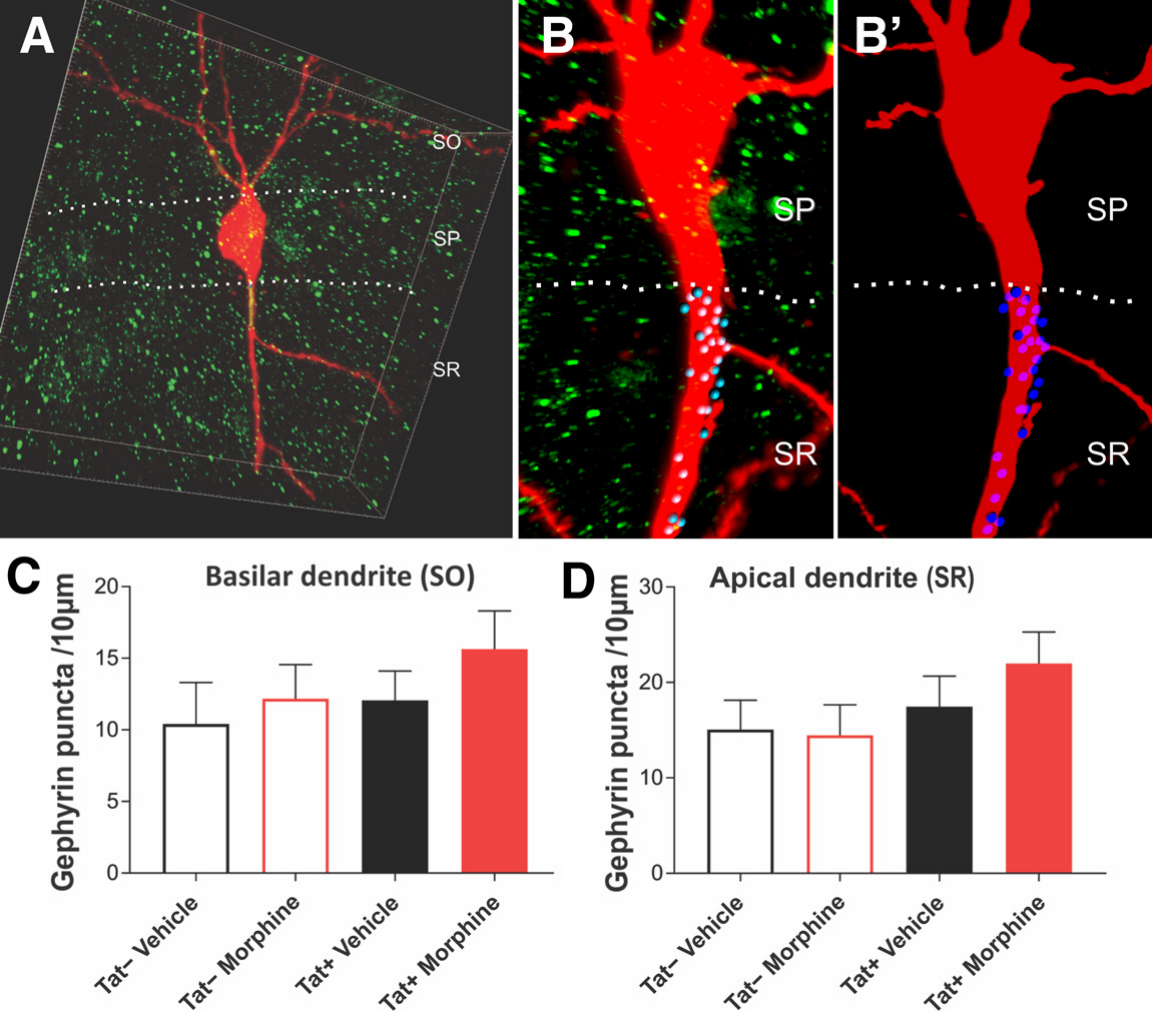
Assessment of inhibitory, postsynaptic gephyrin puncta within the aspinous, proximal dendrites of CA1 pyramidal cells. Biocytin-filled pyramidal cells labeled with an Alexa Fluor 594-conjugated streptavidin probe (red) and gephyrin puncta (green) were analyzed. ***A***, ***B***, ***B’***, A third channel (blue, with darker blue puncta are in front of the dendrite; lighter blue puncta are behind the dendrite; ***B***, ***B’***), only identifying the gephyrin puncta that were adjacent or overlapped with the aspinous portions of the basilar (***C***) or perisomatic apical (***D***) pyramidal cell dendrite, from 3D-reconstructed images was quantified. No changes in the number of gephyrin-immunoreactive puncta were observed. Box Dimensions in A: 100 × 100 × 20 μm. Data represent the mean number of gephyrin-positive puncta ± SEM per 10-μm length of dendrite.

To assess whether Tat-dependent reductions in specific subsets of interneurons (Marks et al., 2016) and Syt2-expressing presynaptic fibers in the SR in CA1 (Fitting et al., 2013) resulted in the loss of inhibitory postsynaptic terminals, the number of puncta was quantified along the aspinous portions of the basilar and apical dendrites of pyramidal cells as they emerged from the soma. This region of both the basilar and apical portions of the dendrite is known to have the highest percentage of inhibitory contacts onto pyramidal cells (Megías et al., 2001). The results show no significant interactions between or main effects of either Tat or morphine on the number of inhibitory puncta within the basilar or apical dendritic shafts in the perisomatic region (Fig. 6).

### CA1 volume was unaffected by morphine and Tat exposure, while Tat increased the volume of CA3

**Figure 7.**
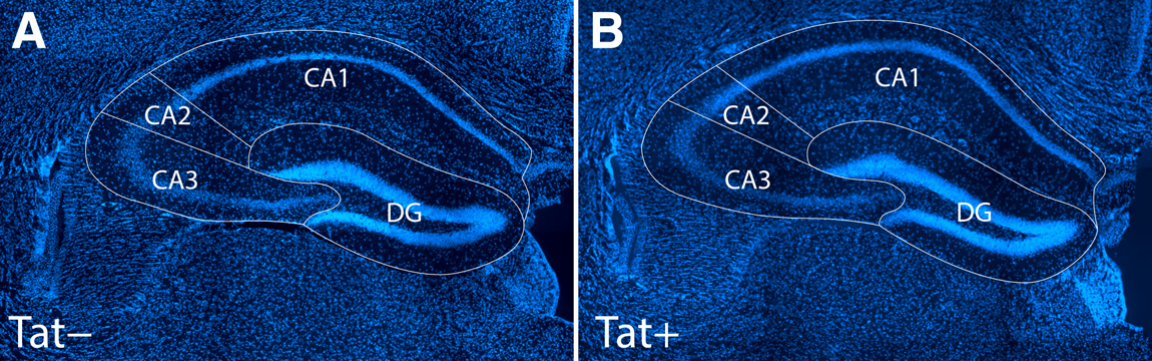
***A***, ***B***, There was no effect of Tat or morphine exposure on the V_V_ of hippocampal areas CA1, CA2, or the dentate gyrus (DG); however, the volume of CA3 was significantly increased by Tat induction regardless of morphine exposure (see Table 1). Importantly, the measurements of dendritic spine density (Figs. 4, 5) were not distorted by morphine-dependent or Tat-dependent alterations in the volume of hippocampal area CA1. Images show representative Hoechst 33342-stained, 40-μm-thick coronal sections of the hippocampus from Tat− (***A***) and Tat+ (***B***) mice used for stereology.

Stereology was performed to assure any changes in dendritic spine density/morphology in CA1 were not distorted by alterations in the volume in this region of the hippocampus (e.g., through gliosis, vascular leakiness (Leibrand et al., 2019); or possible disruptions in glymphatic drainage). No significant influence of morphine or Tat genotype was observed on the V_V_s of CA1, CA2, or the dentate gyrus. By contrast, the volume of CA3 was significantly greater among Tat+, compared with Tat−, mice regardless of whether morphine was administered (*F*_(1,20)_ = 6.350; η^2^ = 0.20; *p *=* *0.02; Fig. 7; Table 2).

**Table 2:** Stereological analyses of hippocampal volume

	Tat−	Tat−	Tat+	Tat+
	Vehicle	Morphine (25 mg)	Vehicle	Morphine (25 mg)
CA1	44.35 ± 2.48%	43.97 ± 0.72%	40.19 ± 3.48%	46.13 ± 3.18%
CA2	5.00 ± 0.71%	5.03 ± 0.68%	4.70 ± 0.65%	5.21 ± 1.07%
CA3	26.71 ± 1.93%	26.58 ± 1.53%	35.92 ± 2.26%*	28.91 ± 2.88%*
DG	26.94 ± 1.45%	24.42 ± 1.24%	28.31 ± 2.19%	28.07 ± 2.87%

No significant alterations in total hippocampal volume fraction (V_v_) for CA1, CA2, or the dentate gyrus (DG) were found in male Tat transgenic mice (*n *=* *5–8) following two weeks of Dox and 5 d subcutaneous vehicle-containing or morphine (25 mg)-containing time-release implants. A significant increase in hippocampal area CA3 volume was detected for Tat+ mice; * indicates a significant difference between Tat− and Tat+ tissues, *p *<* *0.05 and also indicated in bold text.

### Morphine and Tat exposure impaired Barnes maze performance

**Figure 8.**
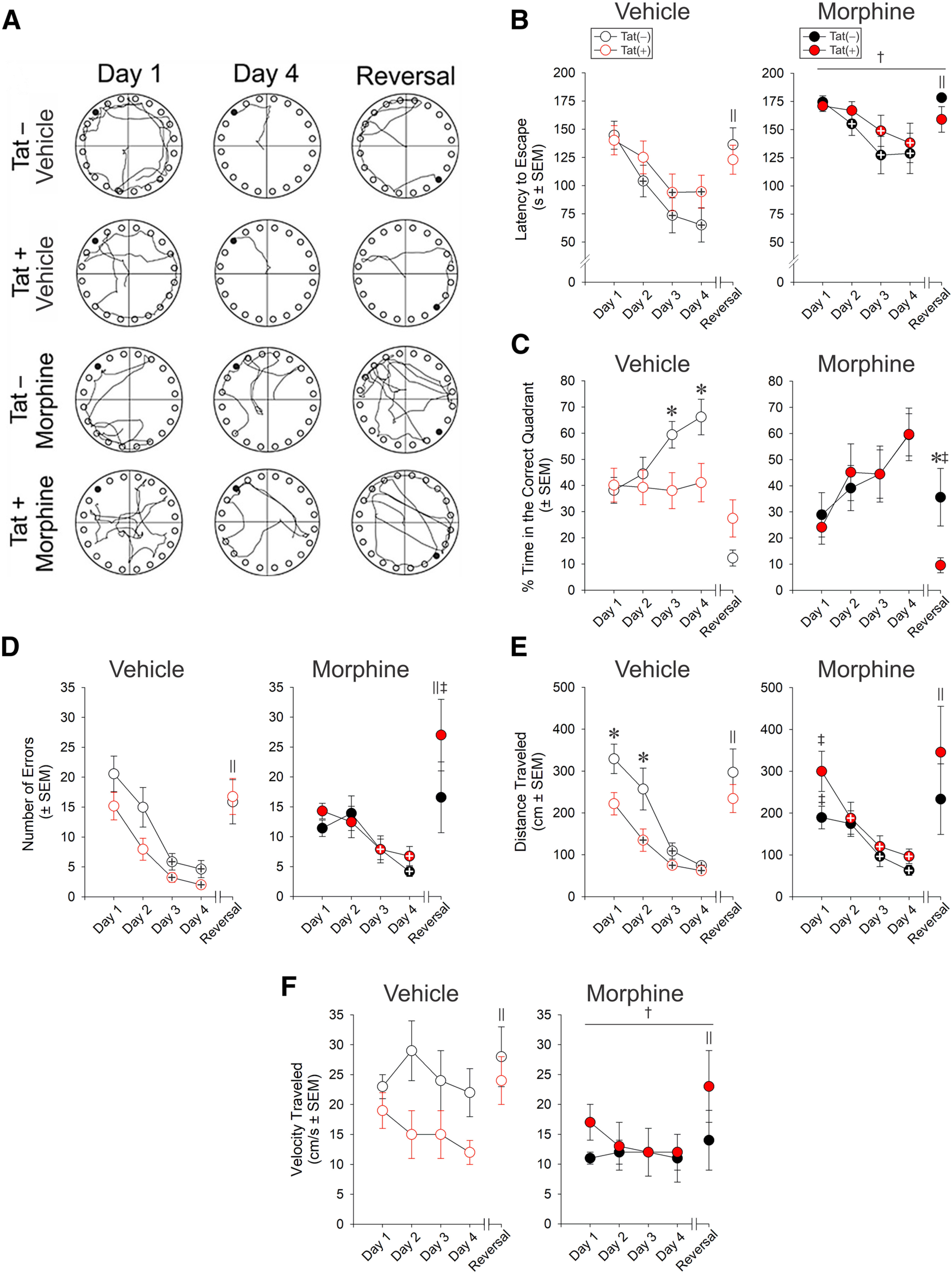
Effects of morphine and HIV-1 Tat exposure on spatial learning. ***A***, Representative paths traveled by vehicle-exposed or morphine-exposed Tat− and Tat+ mice (*n *=* *12–17/group) on the Barnes maze test (the filled black circle indicates the escape goal; open circles indicate decoy escape locations). ***B***, Tat− mice showed a significant decrease in the latency (s) during 4 d of training trials to find the escape hole compared with Tat+ mice, while morphine increased the latency regardless of genotype. ***C***, Tat− mice spent a greater proportion of time in the correct quadrant than Tat+ mice. Although morphine increased the proportion of time Tat+ mice spent in the correct quadrant, morphine-treated mice spent significantly less time in the correct quadrant during the reversal probe trial. ***D***, Tat− and Tat+ mice made fewer errors on days 3 and 4. Morphine increased the errors made by Tat+ mice on the reversal probe trials. ***E***, Tat+ mice traveled shorter distances (cm) on days 1 and 2 and reduced their daily travel significantly sooner than Tat− mice. On day 1, morphine increased the distance traveled by Tat+ mice, while reducing the distance traveled by Tat− mice. ***F***, The velocity of travel (cm/s) was reduced by morphine exposure. ^†^ main effect for morphine differed from vehicle treatments; ^‖^ reversal day performance significantly differs from day 4 performance; ^+^ indicates the group significantly differs from day 1 performance; * indicates the Tat− and respective Tat+ groups differ significantly; ^‡^ indicates the morphine and respective vehicle-treated groups differ significantly, *p *<* *0.05.

Pyramidal neuron firing within the CA1 area of the hippocampus plays an important role in spatial memory (Gothard et al., 1996). Tat− and Tat+ mice, implanted with vehicle-containing or morphine-containing pellets were assessed on the Barnes maze across 4 d and a reversal probe trial (Fig. 8*A*). Compared with those implanted with vehicle pellets, mice implanted with morphine pellets took significantly longer to find the escape hole, regardless of Tat genotype (*F*_(1,216)_ = 15.15, *p *<* *0.05; η^2^ = 0.22; Fig. 8*B*). Genotype significantly interacted with the day of testing (*F*_(4,216)_ = 3.60, *p *<* *0.05; η^2^ = 0.004) such that Tat− mice demonstrated a significantly lower latency to escape on every day compared with their initial performance on day 1 (*p *<* *0.001–0.001; Cohen’s *d *=* *0.7–1.1; Fig. 8*B*). In contrast, Tat+ mice did not outperform their initial day 1 performance until days 3 and 4 (*p *<* *0.001; Cohen’s *d *=* *0.6–0.7; Fig. 8*B*). Both strains of mice demonstrated a significantly increased latency to escape on the reversal probe trial compared with their day 4 performance (*p *<* *0.0001–0.003; Cohen’s *d *=* *0.4–1.0; Fig. 8*B*).

Similarly, there was a significant three-way interaction for the proportion of time spent in the correct quadrant of the Barnes maze (*F*_(4,216)_ = 3.96, *p *<* *0.05; η^2^ = 0.05), such that Tat+ mice spent less time in the correct quadrant than did Tat− controls on days 3 and four of testing (*p *=* *0.02–0.045; Cohen’s *d *=* *0.9; Fig. 8*C*). Morphine did not affect the percent of time spent in the correct quadrant on days 1–4; however, on the day of reversal testing, morphine significantly increased the proportion of time spent in the correct quadrant among Tat− mice (*p *=* *0.01; Cohen’s *d *=* *0.9), while tending to decrease it among Tat+ mice (*p *=* *0.056; Cohen’s *d *=* *0.8; Fig. 8*C*).

The number of errors made (*F*_(1,212)_ = 4.90, *p *<* *0.05; η^2^ = 0.08; Fig. 8*D*) and distance traveled (*F*_(1,216)_ = 9.10, *p *<* *0.05; η^2^ = 0.14; Fig. 8*E*) in the Barnes maze were also significantly influenced by Tat and morphine. Tat+ mice administered morphine made more errors (*p *=* *0.02; Cohen’s *d *=* *0.4; Fig. 8*D*) and traveled greater distances on day 1 (*p *=* *0.02; Cohen’s *d *=* *0.4; Fig. 8*E*) than did their vehicle-exposed counterparts. Conversely, morphine decreased the distance traveled by Tat− controls compared with their vehicle-exposed counterparts (*p *=* *0.04; Cohen’s *d *=* *0.4; Fig. 8*E*). After day 1, all groups made fewer errors (*F*_(4,212)_ = 22.16, *p *<* *0.05; η^2^ = 0.28; Fig. 8*D*) and traveled less distance (*F*_(4,216)_ = 21.91, *p *<* *0.05; η^2^ = 0.28; Fig. 8*E*) on subsequent testing days (*p *<* *0.0001–0.006; Cohen’s *d *=* *0.1–1.1). On the reversal probe trial, all groups made more errors (*p *<* *0.0001; Cohen’s *d *=* *1.2) and traveled a greater distance (*p *< 0.0001; Cohen’s *d *=* *1.1) compared with their day 4 performance. Irrespective of genotype, there was a significant main effect for morphine to reduce the speed at which mice traveled (*F*_(1,216)_ = 6.30, *p *<* *0.05; η^2^ = 0.10; Fig. 8*F*). The speed of travel for Tat+ animals was notably greater on the reversal probe trial compared with any other day of testing (*p *<* *0.0001–0.03; Cohen’s *d *=* *0.3–0.5; Fig. 8*F*).

## Discussion

The interactive effects of morphine and HIV-1 Tat were assessed on the intrinsic and evoked activity of hippocampal neurons. Changes in pyramidal cell structure and function were compared with subsequent behavioral deficits associated with neuroHIV and OUD. Morphine was delivered via subcutaneously implanted pellets, which are widely used to administer sustained, high circulating concentrations in rodents. The 25-mg implants deliver clinically relevant plasma/tissue levels of morphine (Ghazi-Khansari et al., 2006), inducing tolerance (Chefer and Shippenberg, 2009) and physical dependence within 3 d in C57BL/6 mice (Bogulavsky et al., 2009). Unlike the numerous opioid and HIV interactions in the striatum (Fitting et al., 2020), Tat and morphine appear to act largely independently to disrupt hippocampal neuronal function. However, withholding morphine from morphine-dependent tissues during *ex vivo* recordings revealed significant, albeit subtle, opioid-HIV interactions with respect to pyramidal excitability and dendritic plasticity at higher stimulating currents. Additional studies are required to determine the extent to which these responses reveal underlying neuroadaptive changes in response to sustained morphine exposure, and the extent to which the pharmacokinetics of opioid exposure mediate pathophysiological opioid and HIV interactions.

Prior observations of selective reductions in GABAergic interneuronal subpopulations (Marks et al., 2016) and reduced GABAergic SR afferents (Fitting et al., 2013) infer that Tat should, on net balance, increase neuronal excitability, as has been observed *in vitro* (Magnuson et al., 1995). However, Tat exerted the opposite effect at lower current intensities in the present study. This unanticipated decrease in CA1 pyramidal cell firing rates might result from (1) Tat-induced (and presumably transient) excitotoxic increases in GABA release from vulnerable interneurons; (2) compensatory changes in GABAergic interneuronal networks/network oscillations (Fitting et al., 2013, 2020; Marks et al., 2016); (3) Tat-mediated increases in gephyrin, a GABA_A_ receptor organizer (Fitting et al., 2013; Choii and Ko, 2015); and/or (4) altered connectivity from synaptodendritic injury associated with chronic, low-level Tat exposure (Dickens et al., 2017). Tat not only disrupts the organization of excitatory and inhibitory connections impacting pyramidal cells in CA1 (Marks et al., 2016) and elsewhere in the hippocampus (Bruce-Keller et al., 2003; Maragos et al., 2003), it can also adjust inhibitory and excitatory activity through compensatory homeostatic alterations in synaptic scaling (Hargus and Thayer, 2013; Green et al., 2019). Notably, our findings identify more robust Tat-dependent deficits on CA1 pyramidal cell excitability than seen in an alternate HIV-1 Tat transgenic mouse model (Cirino et al., 2020), which may result from more prolonged (14-d) Tat exposure.

### Tat alters long-term circuit neuroadaptations to morphine

In mice maintained on morphine, pyramidal cell responsiveness differed depending on whether morphine was present or withheld during the recordings. Sustained morphine exposure during the recording reduced the firing threshold but did not impact overall firing rates. By contrast, when morphine was withheld during recording, pyramidal cells had significantly lower firing rates at high stimulating currents without a commensurate change in firing threshold. Since acute morphine exposure typically increases CA1 pyramidal cell firing by inhibiting presynaptic afferents from MOR-expressing inhibitory interneurons (Siggins and Zieglgänsberger, 1981), and can hyperpolarize specific subsets of interneurons (Drake and Milner, 2002; McQuiston and Saggau, 2003; McQuiston, 2008), changes in CA1 pyramidal cell excitability following sustained morphine exposure may result from neuroadaptation because of abnormal presynaptic inhibition, changes in ion channel composition or distribution, and/or altered microcircuitry. Abruptly withholding morphine’s inhibitory effects from opioid-tolerant interneurons likely results in their transient overexcitation, excess presynaptic GABA release (Christie, 2008), and the suppression of pyramidal cell firing rates. When morphine was withheld from the slices taken from morphine-pelleted mice, significant reductions were seen in both (1) firing thresholds and (2) firing frequencies at high current amplitudes, although intrinsic resistance and capacitance measures were largely unchanged by Tat and morphine. Neuroadaptation seems to occur at multiple levels, since alterations in the number and morphologic type of dendritic spines along specific dendritic domains were also evident. Our findings are generally consistent with known circuit/ensemble-based effects of morphine on CA1 neuronal activity (Harrison et al., 2002; Liu et al., 2010; Farahmandfar et al., 2011a,b).

Tat additionally reduced intrinsic excitability, which is also seen with prolonged Tat applications (Francesconi et al., 2018). Analysis of contrasts assessing possible morphine and Tat interactions revealed that changes in membrane potential and rheobase were largely driven by Tat. Interestingly, more protracted exposure to Tat does not alter intrinsic membrane properties despite altered excitability (Francesconi et al., 2018), suggesting the network effects of Tat precede its direct effects on CA1 pyramidal cells. However, based on morphologic measures and past studies, Tat is likely affecting both the pyramidal cells and the interneuron network (Fitting et al., 2013; Marks et al., 2016; Francesconi et al., 2018).

### Deficiencies in dendritic spines are associated with the severity of HAND and decreased plasticity

The development of HAND has been associated with HIV-1-induced synaptodendritic injury and culling (Ellis et al., 2007; McArthur et al., 2010; Saylor et al., 2016; Smail and Brew, 2018). Tat-induced reductions of spine density have been observed in the cortex, striatum, and hippocampus (Fitting et al., 2013; Hahn et al., 2015; Raybuck et al., 2017; Schier et al., 2017), while sustained morphine exposure is known to reduce the density of spines in CA1 pyramidal cells (Robinson et al., 2002; Zheng et al., 2010). Within the SO, we found reductions in dendritic spine density with morphine treatment, but not Tat expression. Acute morphine withdrawal also tended to reduce spine densities in the SO and SR. Previously, Tat reduced the overall density of spines by 11% along the apical dendrite in Golgi-impregnated CA1 pyramidal cells (Fitting et al., 2013). The numerical differences between Golgi versus biocytin studies may reflect differences in methodology or focus since spine densities within specific hippocampal layers or morphologic spine subtypes (described below) were not previously studied.

Subtle Tat and/or morphine-dependent rebalancing between excitatory and inhibitory synapses likely occurs within discrete areas of the hippocampal laminar structure, since Tat (Marks et al., 2016) and morphine (Drake and Milner, 1999, 2002) differentially affect distinct interneuronal subpopulations having unique regional distributions. In the SL-M, morphine withdrawal increased the numbers of stubby spines regardless of Tat and decreased thin/filopodial spines along the dendrites of Tat+ mice, indicating rapid alterations in synaptic plasticity along specific regions of the dendrite. Dendritic spine stability is susceptible to inflammation (Beroun et al., 2019) and opiate-induced or HIV-induced alterations in NeuroD signaling (Liao et al., 2005; Zheng et al., 2010; Yuferov et al., 2013). These morphologic changes can occur rapidly, as observed in an animal model of cocaine abuse (Stankeviciute et al., 2014). Mushroom spines, which are considered the most mature/stable form, were selectively reduced in the SR following 14-d Tat exposure. While the SR receives input from Shaffer collaterals, the SL-M receives distinct inputs of differentially processed spatial and non-spatial sensory information from the entorhinal cortex via the temporoammonic and perforant path fibers. The putative integrative mechanism formed by the interneuron network and pyramidal cells in CA1 can easily be unbalanced, leading to memory formation dysfunction (Kitamura et al., 2014, 2015; Marks et al., 2016, 2020). Although our findings show no differences in gephyrin puncta within pyramidal cell dendrites near the soma (Fig. 7), previously demonstrated increases in gephyrin levels do not discriminate pyramidal cells from other CA1 neuron types (Fitting et al., 2013). Selective spine losses in combination with disruptions to the inhibitory interneuron network (Fitting et al., 2013; Marks et al., 2016) are likely to contribute to decreased LTP (Bao et al., 2007; Fitting et al., 2013) and the altered CA1 pyramidal cell structure and function seen here.

### Morphine and Tat disrupt spatial memory

HIV-1 Tat has previously been shown to attenuate spatial memory in the Barnes maze task (Carey et al., 2012; Kesby et al., 2016; Marks et al., 2016). Tat-mediated impairment of spatial memory coincided with reductions in pyramidal cell excitability at lower stimulating currents in the present study. Morphine treatment counteracted some of the negative effects of Tat, such as the proportion of time spent in incorrect quadrants but exacerbated the number of errors made by Tat+ mice on a reversal probe trial and greatly increased the latency for all mice to find the escape hole. These findings may reveal cognitive distinctions between morphine’s effects to potentially preserve aspects of Tat-impaired reference memory, while impeding novel search strategies. The notion that Tat and morphine can independently impair spatial memory is consistent with the minimal behavioral interactions observed in the current study (Zhu et al., 2011; Kitanaka et al., 2015; Kesby et al., 2016; Marks et al., 2016). Opioid-dependent individuals reportedly demonstrate hyper-connectivity in hippocampo-amygdalar memory circuits that may promote the recall of drug-associated cues (Zhang et al., 2017; Ma et al., 2020). This hyperactive associative circuitry may counterbalance the general effects of Tat (Basu and Siegelbaum, 2015; Roy et al., 2017; Sharifi et al., 2020).

The morphine implants used result in relatively high plasma levels, like those seen in OUD in which tolerance to high opioid dosages is typical. Higher morphine doses can generate a biphasic response in which activity oscillates before returning to baseline (Vasko and Domino, 1978). Tolerance to morphine’s depressive effects develops more rapidly than to its stimulatory effects (Vasko and Domino, 1978; Ling et al., 1989; Le Marec et al., 2011), raising the possibility of locomotor confounds. In the present study, morphine treatment reduced overall velocity and initial distance traveled among Tat− mice, but increased distance traveled by Tat+ mice. Differences in the distance traveled were not evident after day 1, but we cannot rule out the contribution of motor influences given the high dose of morphine used in the present study, particularly during the first few days of exposure, while mice are still developing tolerance to the drug.

The collective findings suggest that the structure and function of CA1 pyramidal cells are dramatically influenced by morphine exposure. The presence of an additional stressor such as Tat may enhance the destabilizing effects of fluctuating morphine levels. While the striatum tends to display additive or synergistic interactions, Tat and morphine acted more independently and occasionally seemed to counteract each other in the hippocampus. Rapid alterations in pyramidal cell structure and function in response to opiate withdrawal underscore the potential importance of opiate pharmacokinetics and the sustained consequences of fluctuating drug levels (as seen in OUD) in driving the pathobiology of opiate abuse-neuroHIV comorbidity. The nuanced regional effects of HIV and opioid drugs open exciting questions on the extent to which network level effects on behavioral dysfunction are driven by the unique interplay of pathologic interactions and counteractive processes in distinct brain regions, and whether the counteractive effects can be used as a treatable target.

## Synthesis

Reviewing Editor: Lindsay De Biase, University of California, Los Angeles Decisions are customarily a result of the Reviewing Editor and the peer reviewers coming together and discussing their recommendations until a consensus is reached. When revisions are invited, a fact-based synthesis statement explaining their decision and outlining what is needed to prepare a revision will be listed below. The following reviewer(s) agreed to reveal their identity: NONE. Note: If this manuscript was transferred from JNeurosci and a decision was made to accept the manuscript without peer review, a brief statement to this effect will instead be what is listed below.

In this manuscript transferred from Journal of Neuroscience, the authors use a combination of histology, slice electrophysiology, and behavior to investigate the impact of Tat and morphine on hippocampal function. The experiments are well executed and the data and analysis appear to be of high quality. The authors find distinct effects of Tat exposure and Tat+ morphine omission on CA1 pyramidal neuron excitability, postsynaptic dendritic spines, and behavior. As such, the manuscript is potentially suitable for publication in eNeuro.

However, a concern in reviewer comments was the lack of a cohesive logic or narrative linking the different levels of investigation. This concern remains and we suggest additional changes to further enhance the reader's ability to extract a “take home message” from the data presented.

1) The Stereological analysis presented in figure 1 and table 1 is largely supportive of the validity of structural analyses of dendritic spines in CA1 pyramidal neurons in figures 4 and 5. Hence, it is somewhat confusing to have it presented first. We suggest moving this to after figure 5 and referencing this data after presenting the spine analysis as a good indication that spine analysis results are not influenced by significant changes in hippocampal tissue structure.

2) Additional narrative lead in and justification is needed for each section of the results. For example, at the beginning of the section on electrophysiological properties of CA1 pyramidal cells, the authors should describe why they chose to investigate membrane excitability rather than excitatory and inhibitory synaptic input. Examining excitatory synaptic input (via analysis of mEPSCs for example) would link more naturally to the anatomical analysis of postsynaptic dendritic spines. Examining inhibitory synaptic input (via analysis of mIPSCs for example) would link more naturally to the immunohistochemical analysis of gephyrin. Hence the authors should justify why they chose to focus on membrane excitability in the context of Tat and morphine exposure. Similarly, a brief description of why the authors chose to analyze spine density in different strata of the hippocampus / dendritic arbors will make this manuscript more accessible to readers from diverse areas of expertise. What are the distinct functions of synaptic inputs at each of the strata and what would be the circuit implications of changes in spines within specific strata.

3) In the quantification of different dendritic spine morphologies, the authors state that “about one third of spines” with intermediate or indefinite morphology were excluded. If slightly different numbers/proportions of spines were excluded from each image, how can the authors ensure that this doesn't bias the results? Ideally, the authors would execute this quantification both while excluding these indefinite spines and when including them and assigning them to a specific category to the best approximation. If conclusions are the same in both cases, this strengthens confidence in the results presented.

4) The analysis of inhibitory postsynaptic puncta is completely distinct from the analysis of postsynaptic dendritic spines and should be presented with its own subheading in the text of the results.

5) The term placebo should be replaced with the term vehicle. Because the ex vivo morphine withheld condition is not a true recapitulation of in vivo withdrawal, these should be consistently referred to as “no morphine” or “morphine withheld” throughout the figures and text. In many places, the term “morphine withdrawn” is still used.
